# Biological Activities and Chemical Characterization of *Cordia verbenacea* DC. as Tool to Validate the Ethnobiological Usage

**DOI:** 10.1155/2013/164215

**Published:** 2013-06-02

**Authors:** Edinardo Fagner Ferreira Matias, Erivânia Ferreira Alves, Beatriz Sousa Santos, Celestina Elba Sobral de Souza, João Victor de Alencar Ferreira, Anne Karyzia Lima Santos de Lavor, Fernando Gomes Figueredo, Luciene Ferreira de Lima, Francisco Antônio Vieira dos Santos, Flórido Sampaio Neves Peixoto, Aracélio Viana Colares, Aline Augusti Boligon, Rogério de Aquino Saraiva, Margareth Linde Athayde, João Batista Teixeira da Rocha, Irwin Rose Alencar Menezes, Henrique Douglas Melo Coutinho, José Galberto Martins da Costa

**Affiliations:** ^1^Universidade Estadual do Ceará-UECE-60740-000, Fortaleza, CE, Brazil; ^2^Rede Nordeste de Biotecnologia-RENORBIO-60740-000, Fortaleza, CE, Brazil; ^3^Faculdade Leão Sampaio-CE-FALS-63180-000, Juazeiro do Norte, CE, Brazil; ^4^Universidade Regional do Cariri-URCA-63.100-000, Crato, CE, Brazil; ^5^Universidade Federal do Maranhão-UFMA-65085-580, São Luís, MA, Brazil; ^6^Universidade Federal de Santa Maria-UFSM-97105-900, Santa Maria, RS, Brazil; ^7^Laboratório de Microbiologia e Biologia Molecular, Departamento de Química Biológica, Universidade Regional do Cariri-URCA, Crato-CE, Brasil. Rua Cel. Antonio Luis 1161, Pimenta 63105-000, Brazil

## Abstract

Knowledge of medicinal plants is often the only therapeutic resource of many communities and ethnic groups. “Erva-baleeira”, *Cordia verbenacea *DC., is one of the species of plants currently exploited for the purpose of producing a phytotherapeutic product extracted from its leaves. In Brazil, its major distribution is in the region of the Atlantic Forest and similar vegetation. The crude extract is utilized in popular cultures in the form of hydroalcoholic, decoctions and infusions, mainly as antimicrobial, anti-inflammatory and analgesic agents. The aim of the present study was to establish a chemical and comparative profile of the experimental antibacterial activity and resistance modifying activity with ethnopharmacological reports. Phytochemical prospecting and HPLC analysis of the extract and fractions were in agreement with the literature with regard to the presence of secondary metabolites (tannins and flavonoids). The extract and fraction tested did not show clinically relevant antibacterial activity, but a synergistic effect was observed when combined with antibiotic, potentiating the antibacterial effect of aminoglycosides. We conclude that tests of antibacterial activity and modulating the resistance presented in this work results confirm the ethnobotanical and ethnopharmacological information, serving as a parameter in the search for new alternatives for the treatment of diseases.

## 1. Introduction

In many developing countries, various communities do not have sufficient resources to meet their needs with regard to obtaining medicine to treat various diseases [1]. Thus, these communities often depend on natural resources, including native plant species to fulfill or complement their therapeutic resources [2–4].

Popular observations on the use of medicinal plants contribute in a relevant way to spread awareness of the therapeutic properties of plants often prescribed because of the medicinal effects they exhibit, despite that the chemical constituents of many are not known [5].

In the last years, there has been great scientific interest in chemicals and pharmacological investigations of the biological properties of medicinal plants. Medicinal plants have been the source of many medications that are now applied in clinical practice [6–10]. 

Brazil is the country with the greatest plant genetic diversity in the world, accounting for more than 55,000 cataloged species out of an estimated total of between 350,000 and 550,000 species. Many of these species are endemic to a region and still have not been evaluated from a phytochemical and pharmacological point of view [11].

“Erva-baleeira,” *Cordia verbenacea* DC., is one of the species of plants currently exploited in this sense, for the purpose of producing a phytotherapeutic product extracted from the leaves. The genus *Cordia* belongs to the family Boraginaceae, which includes about 250 species, where the majority have a bush or tree size. The species *C. verbenacea* DC. is native to Central and South America [12]. In Brazil, its greatest distribution is in the region of the Atlantic Forest and low areas of the Amazon [13]. The species can reach up to three meters in height, but when grown as crops in this country, the plants are only one meter high [14].

The crude extract of the aerial parts of the herb (leaves and stems) is widely utilized in popular, medicine in the form of hydroalcoholic extracts, decoctions, and infusions, mainly as antimicrobial, anti-inflammatory, and analgesic agents. Pharmacological studies have demonstrated that products obtained from *C. verbenacea* have a pronounced anti-inflammatory effect with topical and oral administration, associated with low toxicity and an substantial protective effect on the gastric mucosa of rodents [15].

*Staphylococcus aureus* is distributed in nature, as well as being a part of the normal microbiota of the skin and mucosa of animals, including birds. Some specimens of *Staphylococcus* are frequently recognized as etiological agents of opportunistic infections in various animals and humans [16, 17]. Besides causing different types of poisoning, *S. aureus* is the most common etiological agent of purulent infections (e.g., furuncles, carbuncles, abscess, myocarditis, endocarditis, meningitis, pneumonia, and bacterial arthritis) [18]. *Escherichia coli* is one of the principal pathogens responsible for causing infectious diseases in humans. These bacteria are known for producing enterotoxins, whose properties and role in diarrheal disease have been widely investigated. The activity of cytotoxins and their role in human infections have been identified [19–21], mainly in urinary tract infections [22]. *Pseudomonas aeruginosa* is related to one of the main causative agents of hospital infections, such as peritonitis, bacteremia, urinary tract infections, and surgical infections in immunocompetent individuals [23].

Resistance to antibiotics is a growing and worrisome problem in the treatment of many bacterial diseases [16, 24]. For patients with infections, antimicrobial resistance increases morbidity and mortality, while there is a considerable increase in costs for the health institutions [25, 26]. In view of this situation, there is an increase in the need to obtain new drugs with antibacterial properties that are efficient in combating infections [27].

Therefore, the aim of this study was to justify, using *in vitro* experimental models, the utilization of the extract and fraction obtained from the leaves of *C. verbenacea* DC. as an alternative therapeutic agent and source of new isolated substances, correlating with information described in ethnopharmaceutical studies.

## 2. Materials and Methods

### 2.1. Plant Material

Leaves of *Cordia verbenacea* DC. were collected in the municipality of Crato, Ceará, Brazil. The plant material was identified and dried, and pressed specimens were deposited in the Herbario Prisco Bezerra of Universidade Federal do Ceará (UFC), as N° 044171.

### 2.2. Preparation of Methanolic Extracts and Fraction of *Cordia verbenacea* DC

For the preparation of the extracts, leaves were collected which were kept submersed in methanol separately for 72 h; afterward, the extract was filtered and concentrated using a rotary vacuum evaporator (model Q-344B-Quimis, Brazil) and ultrathermal bath (model Q-214M2-Quimis, Brazil). After obtaining the extract, vacuum fractionation was used to extract from fractions. We obtained 9,45 g of methanolic fraction of methanolic Extract of *Cordia verbenacea* DC. (MFMECV) from 44,33 g of methanolic extract of *Cordia verbenacea* DC. (MECV). The solution utilized in the tests was prepared at a concentration of 10 mg/mL, dissolved in dimethyl sulfoxide (DMSO), and then diluted with distilled water to obtain a concentration of 1024 *μ*g/mL, reducing the DMSO concentration lower than 10%, to avoid the toxicity of DMSO.

### 2.3. Phytochemical Prospecting

The phytochemical tests to detect the presence of heterosides, saponins, tannins, flavonoids, steroids, triterpenes, cumarins, quinones, organic acids, and alkaloids were performed according to the method described by Matos [28]. The tests were based on the visual observation of a change in color or formation of precipitate after the addition of specific reagents, and the results for the extract and fractions studied show presence of tannins Flobabens, flavonoids (flavones, flavonols, xanthones, chalcones, auron, Flavonones, leucoanthocyanidins, and catechins), alkaloids, and terpenes.

The phytochemical tests were performed to detect the presence of secondaries metabolics according to the method described by Matos [28] (Table 1). The tests were based on the visual observation of a change in color or formation of precipitate after the addition of specific reagents.

### 2.4. Chemical, Apparatus, and General Procedures

All chemicals were of analytical grade. Methanol, acetic acid, gallic acid, chlorogenic acid, and caffeic acid, were purchased from Merck (Darmstadt, Germany). Quercetin and rutin were acquired from Sigma Chemical Co. (St. Louis, MO, USA). High performance liquid chromatography (HPLC-DAD) was performed with a Shimadzu Prominence Auto Sampler (SIL-20A) HPLC system (Shimadzu, Kyoto, Japan), equipped with Shimadzu LC-20AT reciprocating pumps connected to a DGU 20A5 degasser with a CBM 20A integrator, SPD-M20A diode array detector, and LC solution 1.22 SP1 software.

### 2.5. Quantification of Compounds by HPLC-DAD

Reverse phase chromatographic analyses were carried out under gradient conditions using C_18_ column (4.6 mm × 150 mm) packed with 5 *μ*m diameter particles; the mobile phase was water containing 2% acetic acid (A) and methanol (B), and the composition gradient was 5% of B until 2 min and changed to obtain 25%, 40%, 50%, 60%, 70%, and 100% B at 10, 20, 30, 40, 50, and 80 min, respectively, following the method described byLaghari et al.[29] with slight modifications. The infusion of the leaves of *Cordia verbenacea* was analyzed at a concentration of 10 mg/mL. The presence of five antioxidants compounds was investigated, namely, gallic acid, chlorogenic acid, caffeic acid, quercetin, and rutin. Identification of these compounds was performed by comparing their retention time and UV absorption spectrum with those of the commercial standards. The flow rate was 0.8 mL/min, injection volume 40 *μ*L and the wavelength were 254 nm for gallic acid, 327 nm for caffeic and chlorogenic acids, and 365 nm for quercetin and rutin. The samples and mobile phase were filtered through 0.45 *μ*m membrane filter (Millipore) and then degassed by ultrasonic bath prior to use. Stock solutions of standards references were prepared in the HPLC mobile phase at a concentration range of 0.020–0.200 mg/mL for quercetin and rutin; 0.050–0.250 mg/mL for gallic, caffeic, and chlorogenic acids. The chromatography peaks were confirmed by comparing its retention time to those of reference standards and by DAD spectra (200 to 500 nm). Calibration curve for gallic acid: *Y* = 12760*x* + 1176.4 (*r* = 0.9997); chlorogenic acid: *Y* = 14158*x* + 1074.9 (*r* = 0.9995); caffeic acid: *Y* = 15734*x* + 1727.5 (*r* = 0.9999); rutin: *Y* = 13721 + 1268.4 (*r* = 0.9997); and quercetin: *Y* = 13795*x*  +  1392.6 (*r* = 0.9991). All chromatography operations were carried out at ambient temperature and in triplicate.

### 2.6. Strains

Experiments were performed with clinical isolates of *Escherichia coli* (EC27), *Staphylococcus aureus* 358 (SA358), and *Pseudomonas aeruginosa* (PA03) resistant to as well as to amikacin, neomycin, and gentamicin [26]. The EC-ATCC10536 strain of *Escherichia coli*, the SA-ATCC25923 strain of *Staphylococcus aureus*, and the PA-ATCC15442 strain of *Pseudomonas aeruginosa* were used as positive controls and were maintained on Heart Infusion Agar slants (HIA, Difco). Prior to the assays, the cells were grown overnight at 37°C in Brain Heart Infusion (BHI, Difco).

### 2.7. Drugs

Gentamicin, amikacin, and neomycin were obtained from Sigma Chemical Corp., St. Louis, MO, USA. All of the drugs were dissolved in sterile water before use.

### 2.8. Antibacterial Test (MIC) and Modulation of Antibiotic Activity

MIC (Minimal Inhibitory Concentration) was determined in a microdilution assay [30–32] utilizing an inoculum of 100 *μ*L of each strain, suspended in brain heart infusion (BHI) broth up to a final concentration of 10^5^ CFU/mL in 96-well microtiter plates, using twofold serial dilutions. Each well received 100 *μ*L of each extract solution. The final concentrations of the extracts varied 512–8 *μ*g/mL. MICs were recorded as the lowest concentrations required to inhibit growth. The minimal inhibitory concentration for the antibiotics was determined in BHI by the microdilution assay utilizing suspensions of 10^5^ CFU/mL and a drug concentration range of 2.500 to 2.4 *μ*g/mL (twofold serial dilutions) [30–32]. MIC was defined as the lowest concentration at which no growth was observed. For the evaluation of the extracts as modulators of resistance to the antibiotics, MIC of the antibiotics was determined in the presence or absence of extract (MECV) and fraction (MFMECV) at subinhibitory concentrations (128 *μ*g/mL), and the plates were incubated for 24 h at 37°C. Each antibacterial assay for MIC determination was carried out in triplicate [26, 30–32]. 

### 2.9. Statistical Analysis of Microbiological Results

The results of the tests were done in triplicate and expressed as geometric mean. Statistical analysis was applied to two-way ANOVA followed by Bonferroni posttests using GraphPad Prism 5.0 software.

## 3. Results and Discussion

The search for new drugs derived from natural products has intensified in the last years [33]. Harvey and collaborators [34] reported that drugs from 225 natural sources were in the development phase, and of these, approximately 80% were extracted from plants. The search for medicines and genes from nature has been fostered as a nondestructive use of habitats, which promote human health, as well as supporting economic development and conservation [35].

### 3.1. HPLC Analysis

**Figure 1:**
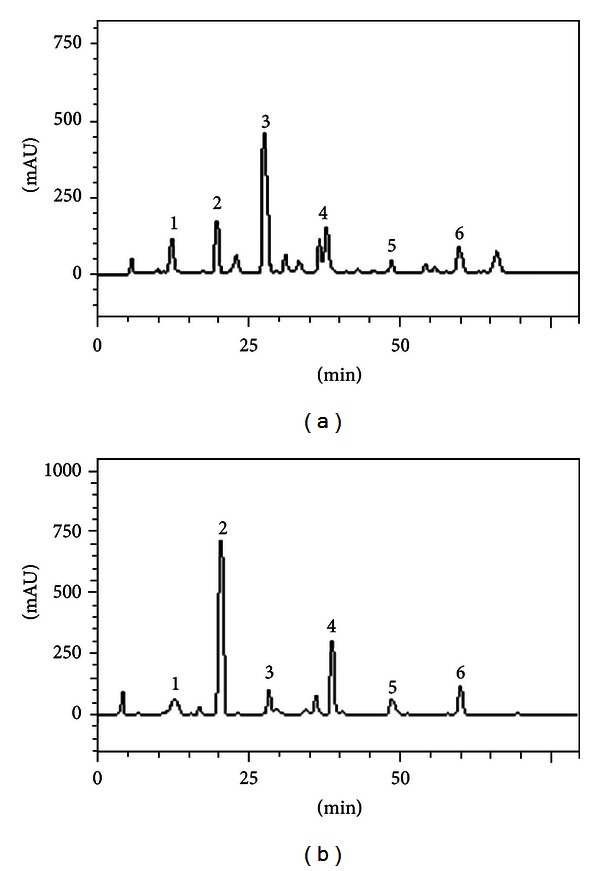
(a) Representative high performance liquid chromatography profile of methanolic extract of leaves *Cordia verbenacea*. Gallic acid (peak 1), chlorogenic acid (peak 2), caffeic acid (peak 3), unidentified glycoside phenol (peak 4), rutin (peak 5), and quercetin (peak 6). Chromatographic conditions are described in Section 2. (b) Representative high performance liquid chromatography profile of methanolic fraction methanolic extract of leaves *Cordia verbenacea*. Gallic acid (peak 1), chlorogenic acid (peak 2), caffeic acid (peak 3), unidentified glycoside phenol (peak 4), rutin (peak 5), and quercetin (peak 6). Chromatographic conditions are described in Section 2.

HPLC fingerprinting of methanolic extract of leaves *Cordia verbenacea* revealed the presence of the gallic acid (*t*_*R*_ = 13.27 min; peak 1), chlorogenic acid (*t*_*R*_ = 20.54 min; peak 2), caffeic acid (*t*_*R*_ = 28.02 min; peak 3), unidentified glycoside phenol (*t*_*R*_ = 39.91 min; peak 4), rutin (*t*_*R*_ = 48.56 min; peak 5), and quercetin (*t*_*R*_ = 60.15 min; peak 6) (Figures 1(a) and 1(b) and Table 2).

Phenolic compounds, including tannins and flavonoids, have demonstrated their therapeutic potential as anti-inflammatory, antifungal, antimicrobial, antioxidant, and wound-healing agents [36].

Some investigators have reported synergism between flavonoids and conventional antibacterial agents against resistant bacterial strains, and others have examined if the activity of flavonoids is bacteriostatic or bactericidal [37]. 

According to Cushnie and Lamb [37], the antibacterial activity of flavonoids has been increasingly more documented. Many researchers are a step further, where they have isolated and identified the structures of commercially available flavonoids, such as rutin, quercetin, 3-O-methylquercetin, and various glycosides of quercetin [38–40]. 

The evaluation of the antibacterial potential of MECV and MFMECV tested against standard and multiresistant strains of *S. aureus*, *E. coli*, and *Pseudomonas aeruginosa* showed a minimum inhibitory concentration of ≥1024 *µ*g/mL for all bacteria strains utilized, where the results were considered clinically irrelevant.

**Figure 2:**
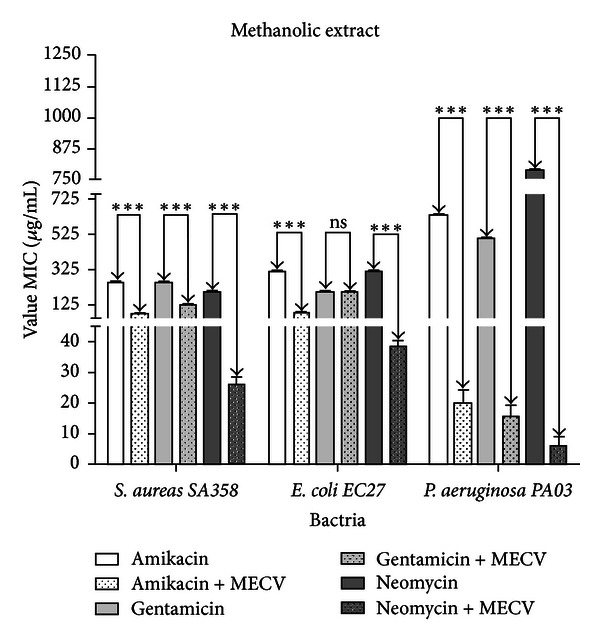
Graph demonstrating the modulatory activity of bacterial resistance to aminoglycoside front of the methanolic extract of *Cordia verbenacea* (MECV). ∗∗∗ value statistically significant with *P* < 0.0001. ns value statistically nonsignificant with *P* > 0.05.

**Figure 3:**
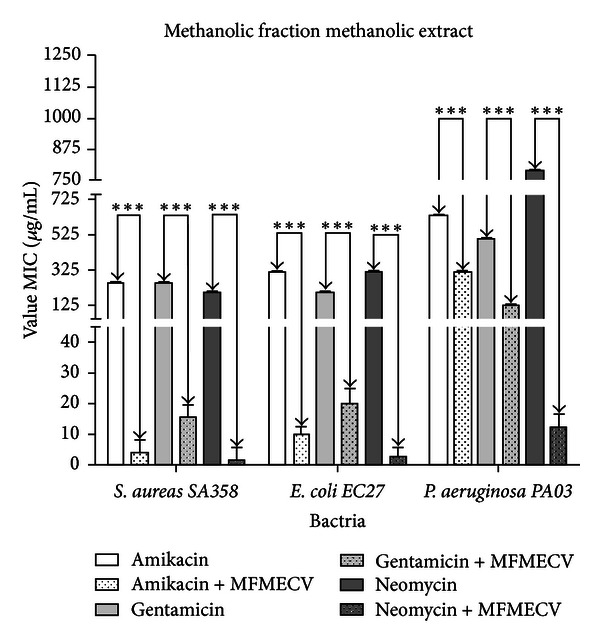
Graph demonstrating the modulatory activity of bacterial resistance to aminoglycoside front of the methanolic fraction of methanolic extract of *Cordia verbenacea* (MFMECV). ∗∗∗ value statistically significant with *P* < 0.0001. ns value statistically nonsignificant with *P* > 0.05.

Figures 2 and 3 showed the results of tests of the modulation of bacterial resistance to aminoglycoside. MECV and MFMECV were found to potentiate the antibacterial effect of the antibiotics tested against all the bacterial strains used, except for MECV when combined with gentamicin and tested against the strain EC27, where there was no statistically significant effect.

 The analysis of Figures 1 and 2 demonstrates that the * S. aureus, E. coli*, and MFMECV exhibit a better activity synergistic combination (antibiotic natural product) compared to the geometric mean MIC, which are statistically significant among themselves. The highest activity in relation to synergistic action might be related to the higher concentration of polar compounds in the fraction. However, analyzing the effect of *P. aeruginosa* is not observed at the same level of modulating action, which could be related to the difference in chemical composition between this and MECV and MFMECV.

The use of extracts as antimicrobial agents presents a low risk of increasing microbial resistance to its action, because they are complex mixtures, providing greater difficulties for microbial adaptability [41].

Despite the search for new substances from plant extracts through their isolation and identification, some results appear to be due to the combination of compounds contained in these complex mixtures, which characterize extracts. We see in some studies that when tested alone these substances demonstrated an antagonistic effect compared with the results of this study, which showed their presence in the extract and fraction by HPLC analysis, but they did not exert their effect when isolated [42].

Comparatively, natural products can differ and have an antibacterial activity or resistance-modifying activity, when considering the existence of differences in polarity and secondary metabolites, which are related to affinities for biological action [43, 44]. The mechanisms by which the extracts and fractions can interfere with the growth of microorganisms are varied and can be due in part to the chemical nature of some components. As a result, they can demonstrate a greater interaction with the lipid bilayer of the cell membrane, affecting the respiratory chain and production of energy [45], or even make the cell more permeable to antibiotics, leading to the interruption of vital cellular activity [46, 47]. These mechanisms of action can be due to the combination of antibiotic with extracts and fractions at a subinhibitory concentration added directly to the culture medium [9, 10]. 

This strategy is called “herbal shotgun” or “synergistic multieffect targeting” and refers to the utilization of plants and drugs in an approach that utilizes combined mono- or multiextracts, which can affect not only a single target but various targets, in which the different therapeutic components act together in a synergistic or antagonistic way. This procedure is not only through the combinations of extracts, but also due to combinations between natural products or extracts and synthetic products or antibiotics [48, 49]. 

The importance of the ethnic knowledge of traditional communities demonstrates that prior ethnobotanical and ethnopharmacological information guide experimental studies *in vitro* and *in vivo* aimed at determining the applicability of popular knowledge in the development of new therapies obtained from phytotherapeutic products as utilized in popular medicine [50].

## 4. Conclusions

Our results indicate that the extract and fraction obtained from leaves of *C. verbenacea* do not possess antibacterial activity that is clinically relevant, but when combined with an antibiotic to evaluate their influence on bacterial resistance to aminoglycosides, the extract and fraction demonstrated significant synergistic activity. The use and sale of products derived from *C. verbenacea* may tend to exert pressure on the populations of this species. Therefore, we recommend the development of management plans for rational and sustainable use of the species, reducing the possible pressure on this species, and more studies with emphasis on the use of the extracts and fractions in the treatment of other diseases.

## Figures and Tables

**Table 1:** Prospecting phytochemistry.

Extract fraction	1	2	3	4	5	6	7	8	9	10	11	12	13	14	15	16
MECV	**−**	**−**	**+**	**−**	**−**	**+**	**+**	**+**	**+**	**+**	**+**	**+**	**+**	**+**	**−**	**+**
MFMECV	**−**	**−**	**+**	**−**	**−**	**+**	**+**	**+**	**−**	**−**	**+**	**−**	**−**	**+**	**−**	**+**

1: phenols; 2: tannins pyrogallics; 3: tannins flobatenics; 4: anthocyanins; 5: anthocyanidins; 6: flavones; 7: flavonols; 8: xanthones; 9: chalcones; 10: auron; 11: flavonons; 12: leucoanthocyanidins; 13: catehins; 14: flavonones; 15: alkaloids; 16: terpenes; +: presence;−: absence; MECV: methanolic extract *Cordia verbenacea*; MFMECV: methanolic fraction methanolic extract *Cordia verbenacea*.

**Table 2:** Phenolics and flavonoids composition of methanolic extract (MECV) and methanolic fraction of methanolic extract (MFMECV) of leaves *Cordia verbenacea. *

Compounds	*Cordia verbenacea *	
	MeOH	
	MECV%	MFMECV%
Gallic acid	1.14 ± 0.01	0.72 ± 0.05
Chlorogenic acid	1.59 ± 0.03	5.74 ± 0.02
Caffeic acid	3.85 ± 0.05	0.53 ± 0.02
Glycoside phenol*	1.43 ± 0.02	2.28 ± 0.01
Rutin	0.38 ± 0.01	0.70 ± 0.03
Quercetin	1.09 ± 0.06	0.77 ± 0.04

Results are expressed as mean ± standard deviations (SD) of three determinations. *quantified as caffeic acid.
